# *NDST1* Preferred Promoter Confirmation and Identification of Corresponding Transcriptional Inhibitors as Substrate Reduction Agents for Multiple Mucopolysaccharidosis Disorders

**DOI:** 10.1371/journal.pone.0162145

**Published:** 2016-09-22

**Authors:** Ilona Tkachyova, Xiaolian Fan, Anne-Marie LamHonWah, Bohdana Fedyshyn, Ingrid Tein, Don J. Mahuran, Andreas Schulze

**Affiliations:** 1 Genetics and Genome Biology, Research Institute, Hospital for Sick Children, Toronto, Ontario, Canada; 2 Department of Laboratory Medicine and Pathobiology, University of Toronto, Toronto, Ontario, Canada; 3 Department of Pediatrics, University of Toronto, Toronto, Ontario, Canada; Augusta University, UNITED STATES

## Abstract

The stepwise degradation of glycosaminoglycans (GAGs) is accomplished by twelve lysosomal enzymes. Deficiency in any of these enzymes will result in the accumulation of the intermediate substrates on the pathway to the complete turnover of GAGs. The accumulation of these undegraded substrates in almost any tissue is a hallmark of all Mucopolysaccharidoses (MPS). Present therapeutics based on enzyme replacement therapy and bone marrow transplantation have low effectiveness for the treatment of MPS with neurological complications since enzymes used in these therapies are unable to cross the blood brain barrier. Small molecule-based approaches are more promising in addressing neurological manifestations. In this report we identify a target for developing a substrate reduction therapy (SRT) for six MPS resulting from the abnormal degradation of heparan sulfate (HS). Using the minimal promoter of NDST1, one of the first modifying enzymes of HS precursors, we established a luciferase based reporter gene assay capable of identifying small molecules that could potentially reduce HS maturation and therefore lessen HS accumulation in certain MPS. From the screen of 1,200 compounds comprising the Prestwick Chemical library we identified SAHA, a histone deacetylase inhibitor, as the drug that produced the highest inhibitory effects in the reporter assay. More importantly SAHA treated fibroblasts expressed lower levels of endogenous NDST1 and accumulated less ^35^S GAGs in patient cells. Thus, by using our simple reporter gene assay we have demonstrated that by inhibiting the transcription of *NDST1* with small molecules, identified by high throughput screening, we can also reduce the level of sulfated HS substrate in MPS patient cells, potentially leading to SRT.

## Background

### MPS and Current Therapies

The Mucopolysaccharidoses (MPS) are a group of inherited lysosomal storage disorders (LSD) in which glycosaminoglycans (GAGs) present as the primary accumulated substance within the lysosomes of many tissues. Degradation of GAGs requires twelve lysosomal enzymes (Table 1). Deficiency in any of these enzymes produces clinical phenotypes that vary from mild to severe forms, with the most severe exhibiting progressive delay in cognitive and motor development, aberration in bone morphogenesis, organomegaly, and cardiovascular abnormality. The more attenuated forms of MPS present with milder symptoms and may remain undiagnosed until adulthood [1].

**Table 1:** Mucopolysaccharidoses. ^1^

Disease	Clinical phenotypes	Enzyme deficiency	Stored substrate	Prevalence^2^
**MPS I**	Hurler	α-L-iduronidase	DS, HS^3^	1 in 88,000
	Hurler/Scheie			
	Scheie			
**MPS II**	Hunter	Iduronate-2-sulphatase	DS, HS	1 in 136,000
**MPS IIIA**	Sanfilippo A	Heparan-N-sulphatase	HS	1 in 114,000
**MPS IIIB^4^**	Sanfilippo B	α-N-Acetylglucosaminidase	HS	1 in 211,000
**MPS IIIC**	Sanfilippo C	Acetyl CoA:N-acetyltransferase	HS	1 in 1,407,000
**MPS IIID**	Sanfilippo D	N-acetylglucosamine-6-sulphatase	HS	1 in 1,056,000
**MPC IIIE^3^**	Sanfilippo E	Arylsulfatase G	HS	n/a
**MPS IVA**	Morquio A	Galactose 6-sulphatase	KS	1 in 169,000
**MPS IVB**	Morquio B	β-Galactosidase	KS	n/a
**MPS VI**	Maroteaux-Lamy	N-Acetylgalactosamine-4-sulfatase	DS	1 in 235,000
**MPS VII^4^**	Sly	Β-Glucuronidase	CS,DS,HS	1 in 2,111,000
**MPS IX**	n/a	Hyaluronoglucosaminidase-1	HA	n/a

n/a–data not available

^1^ Neufeld, E. F. and Muenzer, J., The Mucopolysaccharidoses. In: Scriver, C.R., Beaudet, A.L., Sly, W.S., Valle, D. (ed.): The metabolic and molecular bases of inherited diseases. New York: McGraw-Hill Co., 2001, 3421–3452

^2^ The prevalence of Mucopolysaccharidosis diseases was obtained from Lysosomal Diseases Australia (*www.lda.org.au/about.html)*

^3^ Kowalewski, et al. Proc Natl Acad Sci (USA) 109 (2012) 10310–10315

^4^ Accumulated substrate levels would not be affected by reduced NDST1 activity

Current treatments for MPS primarily rely upon bone marrow transplantation (BMT) and enzyme replacement therapy (ERT). BMT has been shown to be successful as a treatment for the severe form of MPS I, Hurler, if performed early, before maturation and closure of the child’s blood-brain barrier (BBB) [2]. Affected MPS I patients who underwent BMT have shown slower cognitive decline and improvement in clearance of the upper airways, movement, joint stiffness, and hearing. Unfortunately, BMT has very little effect in the treatment of other MPS, especially those with primarily neurological manifestation. Furthermore, despite the improvements in the BMT procedure, the rates of lethality for this treatment remain high [3]. ERT has been developed for patients having visceral forms of MPS I, MPS II, MPS IV, and MPS VI. ERT is also being developed for MPS VII [3;4]. However, despite some success in treating several of the MPSs, neither BMT nor ERT is effective in treating MPS with primarily neurological manifestation, because access of the enzymes derived from bone marrow or the recombinant enzymes used in ERT is precluded by the BBB. As alternative approaches to BMT and ERT, enzyme enhancement therapy (EET) and substrate reduction therapy (SRT) have been developed for several MPSs. These therapies utilize small molecules that often have the capacity to cross the BBB and act in the central nervous system of affected MPS patients. In EET, small molecules function in the endoplasmic reticulum as pharmacological chaperons, assisting the mutant proteins to fold correctly, which protects them from degradation by the endoplasmic reticulum associated degradation system and facilitates their delivery to lysosomes. EET is currently being used and/or investigated for Fabry, Gaucher type I, Pompe, GM1/GM2 gangliosidosis, and MPS IIIC [5]. In SRT, small molecules act by directly inhibiting enzymes involved in the biosynthesis of the accumulated substances. Presently SRT is approved for the treatment of Gaucher type I [6;7] and Niemann Pick type C diseases [8]. Other novel strategies use fusion enzymes able to cross the blood brain barrier, as well as gene therapy for Sanfilippo with several clinical trials to start soon.

We have explored the possibilities of SRT for several MPSs resulting from abnormal degradation of heparan sulfate (HS). In principal, any enzyme contributing to HS precursor polymerization or post-synthetic modification can be a target for SRT. In this study, we identify one isoenzyme N-deacetylase/N-sulfotransferase 1 (NDST1) as a target for developing SRT for MPS I, MPS II, MPS IIIA, MPS IIIC, MPS IIID, and MPS IIIE. The combined prevalence of these six diseases represents ~1 in 34,000 live births (Table 1).

### NDST1

NDST1 belongs to a group of four bi-functional Golgi residing HS modifying enzymes (NDST1-NDST4), which perform the first modification step during HS biosynthesis/maturation. HS chains are synthesized by a number of glycosyltransferases that transfer repeating disaccharide units of N-acetylglucosamine (GlcNAc)-glucuronic acid onto various serine residues on the core protein through tetrasaccharide linker to form a HS precursor. After HS precursor formation, NDSTs remove acetyl groups from selected GlcNAc residues and replace them with sulfate groups using high-energy sulfate donor 3’-phosphoadenosine 5’-phosphosulphate molecule to produce N-sulfo-D-glucosamine. This modification is believed to be required for the further maturation of the nascent HS chain by C5-epimerase (to convert glucuronic acid to iduronic acid) followed by sulfotransferases 2-O-sulfotransferase, 6-O-sulfotransferase, and 3-O-sulfotransferase, which complete the final modifications of HS by addition of 2-O-sulfo groups to iduronic acid and glucuronic acid, and 6-O-sulfo and 3-O-sulfo groups to N-acetyl glucosamine or glucosamine N-sulfate. Thus the level of NDST activity is believed to ultimately define the structure of the mature HS polysaccharide; i.e., the size and frequency of iduronic acid-containing domains with a high content of N- and O-sulfated sugar residues (S-domains) in HS [9;10].

There are 4 NDST isoenzymes, NDST1-4, which evolved from a common ancestral gene and then diverged into two subtypes, NDST3/4 and NDST1/2 [11]. The major isozymes in most tissues and the brain are NDST1 and 2, which share a ~70% sequence identity [11;12]. Systemic inactivation of NDST1 in mice produces reduced N-sulfation levels in the HS polysaccharides in most of their tissues and animals succumb perinatally as a result of lung failure or cerebral hypoplasia. In contrast, NDST2 null mice are viable and their tissue HS appear to be unaffected [11]. However, they exhibit selective defects in their connective tissue-type mast cells [13]. Therefore of the 4 NDST isozymes, the activity of NDST1 is believed to be the most important to target in order to reduce the levels of S-domains in the HS polysaccharides throughout the body and the brain.

## Materials and Methods

### *NDST1* promoter—renilla luciferase-reporter gene constructs

The renilla luciferase reporter constructs containing either *NDST1* promoter, GAPDH promoter (a positive control) or one of two random genomic DNA fragments, RO1 and RO2 (negative controls) in pLightSwitch_Prom expression vector were purchased from SwitchGear Genomics (switchgeargenomics.com). The actual *NDST1* genomic DNA sequence cloned in front of the renilla luciferase consisted of 660 bp upstream and 401 bp downstream of exon 1 (mRNA sequence from the UCSC genome browser: AB209107). The NCBI sequences of the reference human genome for the *GAPDH* promoter corresponded to NG_007073.2, for RO1 –NG_013083.1 and for RO2 –NW_004078000.

### *NDST1* promoter—firefly luciferase-reporter gene construct

The firefly luciferase reporter construct containing *NDST1* promoter was generated by sub-cloning of the promoter fragment from pLightSwitch_Prom vector into pGL4.22 [luc2CP/Puro] vector (Promega). The *NDST1* promoter in pLightSwitch_Prom and the pGL4.22 [luc2CP/Puro] vectors were digested with XhoI and BglII restriction enzymes overnight at 37 ^0^C. Digested products were separated on a 1% agarose gel in TBE (90 mM Tris, 90 mM Boric acid, 2 mM EDTA) and purified by using QIAquick Gel Extraction Kit (Qiagen). The ligation of the *NDST1* promoter fragment into the digested pGL4.22 [luc2CP/Puro] was performed by using T4 DNA ligase (New England BioLabs). Clones containing the *NDST1* promoter insert were obtained by transformation of the overnight ligation reaction into DH5α competent cells (Invitrogen). Transformation procedure and selection of the *NDST1*-positive clones were accomplished by following the protocol supplied by Invitrogen. The integrity of *NDST1* promoter in pGL4.22 [luc2CP/Puro] vector was confirmed by the restriction digestion with XhoI and BglII and by the sequencing, using specific primers for pGL4.22 vector (ACGT Corp).

### Cell cultures and transfections

Primary skin fibroblasts derived from patients with MPS IIIA and MPS IIIC, or an unaffected individual (normal human fibroblast) were provided by Coriell Institute’s Cell Repository (Camden, NJ, USA, https://catalog.coriell.org/) and maintained by the Hospital for Sick Children Tissue Culture Facility. Cell lines were normally maintained in AMEM containing 10% fetal bovine serum (FBS), 100 units/mL penicillin, and 100 μg/mL streptomycin at 37°C in 5% CO_2_. All reagents were purchased from Wisent. Transient transfections were performed according to the Invitrogen protocol. In brief, the day before transfections, HeLa cells were seeded into white 96-well plate (BD Falcon) (4 x 10^4^ cells per well). Then, cells were incubated with 200 ng of *NDST1*, *GAPDH*, RO1 and RO2 renilla luciferase reporter constructs and 0.5 μL of Lipofectamine 2000 (Invitrogen) in antibiotic-free culture medium. The pmaxGFP vector(Lonza) was used as a positive control to determine the transfection efficiency.

### Establishment of the *NDST1* promoter-renilla luciferase expressing permanent cell line

To obtain a permanent cell line stably expressing firefly luciferase under control of *NDST1* promoter, HeLa cells were transfected with 200 ng of the *NDST1* promoter in pGL4.22 [luc2CP/Puro] vector. The pGL4.22-*NDST1* promoter DNA was incubated with 0.5 μL of Lipofectamine 2000 in antibiotic-free culture medium and added to the 4 x 10^4^ cells for 24 hours. The next day cells were passaged at 1:50 dilution into fresh AMEM containing 10% FBS, 100 units/mL penicillin, 100 μg/mL streptomycin and 3 μg/mL puromycin (for selection of transfected cells). At three weeks post-transfection, clonal populations of surviving cells were validated for the expression of firefly luciferase activity.

### Renilla luciferase activity assay

Renilla luciferase activity was determined eighteen hours post-transfection using the LightSwitch Luciferase Assay reagent according to the protocol supplied by SwitchGear Genomics. In brief, after removing media, 100 μL of PBS was added into each well containing transfected cells. Then, 100 μL of Assay Solution containing lysis buffer and renilla luciferase substrate was added directly to each sample well. Plates were left for 30 min incubation before reading. Luminescence readings were performed using Envision 2102 Multilabel Reader (Perkin-Elmer).

### Firefly luciferase activity assay

HeLa cells stably expressing *NDST1* promoter were seeded into white 96-well plate (4 x 10^4^ cells per well) for 16 hours at 37°C in 5% CO_2_. The next day, cells were washed 2 x with PBS and lysed with 20 μL of the lysis buffer (1% Triton-100, 20 mM Tricine). Following lysis, 100 μL of an in-house firefly luciferase assay reagent (20 mM Tricine pH 7.8, 4 mM MgSO_4_, 0.1 mM EDTA, 33.3 mM DTT, 270 μM CoA lithium salt, 530 μM ATP, 470 μM D-luciferin sodium salt) was added into each well. Luminescence produced by cells was measured using Envision 2102 Multilabel Reader (Perkin-Elmer).

### Hexosaminidase assay

The lysosomal hexosaminidase (Hex) assay was used as a control for variability in cell number in transient transfections and as a measure for toxicity in high-content screening (HTS). Luminescence values produced by renilla or firefly luciferase were normalized to fluorescence generated by Hex. The Hex assay was performed in clear 96-well plate (BD Falcon). After measuring luciferase activity, 20 μL of cell lysate was transferred into the well of 96-well plate containing 5 μL of CP buffer (0.1 M citric acid, 0.2 M Na2HPO4, pH 4.1) supplemented with 0.5% HSA. The plate was equilibrated at 37°C for 10 minutes followed by the addition of 25 μL of 3.2 mM of the fluorogenic Hex substrate MUG (4-methylumbelliferyl-2-acetamide-2-deoxy-b-D-glycopyranoside). The reaction was carried out at 37°C for 30 minutes and then terminated by addition of 200 μM MAP, pH 10.5 (0.1 M 2-amino-2-methyl-1-propanol, 40 mM HCl). Fluorescence readings were obtained at 365 nm excitation and 450 nm emission using Molecular Devices Spectra Max M2.

### RNA extraction

Total RNA was extracted from cells using the RNeasy Mini Kit (Qiagen, Maryland, USA) according to the manufacturer’s instructions. Briefly, 600 μL of RLT buffer was added to PBS washed cells from one 10 cm plate. Lysed cells were scrapped off the plate, transferred into an Eppendorf tube and vortexed to homogeneity. After addition of 70% ethanol (1/1, v/v), the cell lysate was transferred into RNeasy spin column. Following several washes using Qiagen supplied buffers, total RNA was eluted from the column with a 30 μL of RNase-free water. Extracted RNA was stored at -80°C.

### Reverse transcription

Complementary DNA (cDNA) was prepared using the QuantiTect Reverse Transcription Kit (Qiagen, Hilden, Germany). Genomic DNA from a total RNA sample was removed by incubation of 1 μg of RNA with 2 x gDNA Wipeout buffer (final volume 14 μL) at 42°C for 2 minutes. According to the manufacturer’s instructions, cDNA was generated by incubation of gDNA-free RNA with reverse-transcription master mix containing Quantiscript Reverse Transcriptase and RT Primer Mix (total volume reaction 20 μL) at 42°C for 30 minutes. Quantiscript Reverse Transcriptase was inactivated by incubation of the sample at 95°C for 3 minutes. cDNA was stored at -20°C.

### PCR analysis

PCR was performed using One*Taq* 2 x Master Mix with GC buffer and One*Taq* High GC Enhancer (New England BioLabs). For each reaction in a final volume of 50 μL, 4 μL of cDNA was mixed with 1 x One*Taq* 2 x Master Mix with GC buffer, 20% of One*Taq* High GC Enhancer, 0.2 μM of each NDST1 primer and 0.05 μM of each 18S rRNA primer. Reactions were subjected for amplification in iCycle Bio-Rad using following thermocycling conditions: 4 minutes at 94°C, followed by 40 cycles at 30 seconds at 94°C, 60 seconds for 55°C (P_B_ region) or 59°C (P_A_ and P_C_ regions), and 2 minutes at 68°C. The primers for *NDST1* gene at P_A_, P_B_, and P_C_ regions were designed by Primer Designer software (Sci Ed Central) and the sequences were TAATA-TAGGCGCTGGGCCGAGGA (P_A_ forward), CCTCCTGTCTCTTGAGTATC (P_B_ forward), GGATTAGGTGCAGCCGTGTT (P_C_ forward), and CTGCCTGCACAGGCTTGAGT (common for all P_A_, P_B,_ P_C_ reverse). The primers for 18S rRNA (NR_003286.2) were CGAACGAGACTCTGGCATGCTAACT (forward) and TCCTTCCGCAGGTTCACCTACG (reverse).

### qPCR analysis

qPCR was performed using iQ SYBR Green Real-Time PCR Supermix from BioRad. For each reaction in a final volume of 10 μL, 2 μL of cDNA was mixed with 1 x IQ^TM^ SYBR Green Supermix and 50 ng of each primer. Reactions were subjected for amplification in MiniOpticon (BioRad) using following thermocycling conditions: 3 minutes at 95°C, followed by 40 cycles at 20 seconds at 95°C, 10 seconds at 52°C, and 20 seconds at 72°C, melting curve 65°C to 95°C, read every 1°C. All reactions were performed in duplicates. The primers for *NDST1* gene were designed by Primer Designer software (Sci Ed Central) and the sequences were TCCTGGTGGACATTGATGAC (forward) and GTGTGCGCGTAGTTCGTTCT (reverse). The primers for 18S rRNA (kindly provided by Dr. Jessie Cameron, Hospital for Sick Children) (internal control) were TAGAGGGACAAGTGGCGTTC (forward) and CGCTGAGCCAGTCAGTGT (reverse). The efficiency of primers was determined by qPCR using serially diluted cDNA from normal human fibroblast cells. For the expression of *NDST1* gene by qPCR analysis, the level of amplified fragments in each reaction was normalized to the level of 18s rRNA. Quantification of relative gene expression was calculated using the 2^–DDC^_T_ (Livak) method.

### Cell treatment for high-content screening (primary screen)

HeLa cells stably expressing *NDST1* promoter—reporter gene construct (8.75 x 10^3^ cells per well) were seeded into a solid white 96-well plate for 24 hours. The next day, 2 μL of each Prestwick Chemical Library^®^ (Prestwick Chemical, San Diego, CA, USA) compound (final concentration 100 μM in 1% DMSO) was added into 80 wells (A2- G11) and 2 μL of DMSO (as a blank) into 16 wells (A1-G1 and A12-G12 rows) of each plate. Following an overnight treatment at 37 ^0^C in 5% CO_2_, the compound-containing media was aspirated and cells were washed 2 x with PBS. Immediately after washing, 20 μL of lysis buffer was added to each well, followed by 30 seconds incubation at room temperature with rocking on a platform shaker. After lysis, 100 μL of the in-house firefly luciferase assay reagent was added. Luminescence readings were collected using Envision 2102 Multilabel plate reader (Perkin-Elmer). All compounds of the Prestwick Chemical Library^®^ were screened in a 96-well format (80 compounds per plate) in triplicates. DMSO was used as a negative control. Variation within a sample set (one plate) was tested by calculating the standard deviation of the average DMSO treatment.

### *In vitro* evaluation of the *NDST1* repressors (‘hits’ from the primary screen)

Specificity of the potential *NDTS1* repressors was evaluated in an *in vitro* firefly luciferase binding assay in triplicates. One confluent 10 cm plate of HeLa cells stably expressing the promoter—reporter construct (8.6 x 10^6^ cells per well) was lysed in 1.7 mL of the lysis buffer. Then, 20 μL of the cell lysate was mixed with 2 μL of 1 mM compound stock in a solid white 96-well plate and left rocking on a platform shaker for 10 minutes at room temperature. Following incubation, 100 μL of in-house firefly luciferase assay reagent was added and the emitted light was measured using Envision 2102 Multilabel plate reader (Perkin-Elmer).

### Generation of dose-response curves for each ‘hit’

Selected compounds, ‘hits‘, from the Prestwick Chemical library were diluted in DMSO to obtain 0.011, 0.033, 0.1, 0.3, 0.9, 2.7, and 8.1 mM stock concentrations. Twenty-four hours prior to the treatment, 8.75 x 10^3^ of the promoter—reporter transfected HeLa cells were seeded into the wells of a white 96-well plate. The following day, compounds at final concentrations of 0.11 μM, 0.33 μM, 1 μM, 3 μM, 9 μM, 27 μM, 81 μM, were added to each well and left for incubation overnight at 37 ^0^C. After the treatment, luminescence response was detected as described above.

### Validation of selected compounds on the endogenous NDST1 protein expression by Western blot

The effect of selected compounds from the Prestwick library on endogenous NDST1 protein level in Hela cells was validated by Western blot analysis. After 5 days treatment at either 1 x EC_50_ or 2 x EC_50_, cells from one 10 cm plate were harvested by scraping and solubilized in 300 μL of lysis buffer (1% Triton-100, 50 mM Tris pH 7.4, 150 mM NaCl) by vortexing. The insoluble cellular remains were separated from the lysates by centrifugation at 13,000 rpm for 1 minute. Before loading on SDS-PAGE gel, samples were prepared by adding 4 x sample buffer (62.5 mM Tris pH 6.8, 10% glycerol, 2% SDS, 0.002% bromophenol blue, 5% β-mercaptoethanol) to 30 μg of total lysate proteins and incubating for 15 minutes at 56°C. Proteins were separated by SDS-PAGE using Nu-PAGE 4–12% Bis-Tris gel (Invitrogen) and electrophoretically transferred to the polyvinylidene fluoride (PVDF) membrane (Bio-Rad) in transfer buffer (10 mM CAPS pH 11, 10% methanol) for 1 hour at 4 ^0^C. After transfer, the membrane was blocked with 5% skim milk in TBS-T (25 mM Tris pH 7.4, 137 mM NaCl, 2.7 mM KCl, 0.1% Tween-20) overnight at 4 ^0^C. After blocking, the membrane was probed with the primary antibodies for 1.5 hour at room temperature and the secondary antibodies for 1 hour at room temperature after washing with TBS-T. Signals were produced using enhanced chemiluminescence (ECL) (Amersham GE Healthcare). The primary antibodies used in Western blot analysis were mouse monoclonal NDST1 (E-9) antibody (Santa Cruz) at 1:200 dilutions, and rabbit polyclonal GAPDH antibody at 1:10,000 dilutions. The secondary antibodies were either horseradish peroxidase conjugated goat anti-mouse (Santa Cruz) or horseradish peroxidase conjugated donkey anti-rabbit (Jackson Immunology) at 1:10,000 dilutions. Films were developed using SRX-101A Medical Film Processor (Konica Minolta Medical & Graphic Inc.).

### Quantitation of the endogenous NDST1, Actin and GAPDH by densitometry analysis

NDST1 expression difference in treated versus untreated cells was assessed using lane profile plots generated by ImageJ one-dimensional electrophoretic gel analysis. Peak areas for NDST1, Actin and GAPDH bands, obtained by the ImageJ Western blot analysis, were converted into percentages and were plotted using relative ratios of samples treated with the compound to the sample treated with DMSO alone.

### Total NDST1-4 enzyme activity by an ELISA assay

A streptavidin-coated plate was washed with TBS-T (10 mM Tris-HCl buffered saline with 0.05% Tween-20, pH 7.4). Then 100 μL of K5 (a bacterial polysaccharide with a structure identical to the nascent, unsulfated HS chain)-biotin, which was diluted to 1.3 μg/mL with 0.2% gelatin in TBS-T, was added to each well and incubated at 37°C for 30 minutes. After washing with TBS-T, 50 μL of a cell lysate from either yeast expressing mouse NDST1 (kindly provided by Dr. Jian Liu from University of North Carolina) (positive control) or treated and untreated normal human fibroblasts, were diluted with MES buffer (50 mM MES, 1% Triton X-100, 10 mM MnCl_2_, pH 6.5) containing 0.2% gelatin, and added to each well. The enzyme reaction was carried out at 37°C for 30 minutes and stopped by adding 5μl 5M NaCl to each well. After completion of the enzyme reaction, the wells were washed and 100 μl of the monoclonal antibody JM403 (Seikagaku Corporation), which recognizes GlcNH_3_ residues produced in the K5 polysaccharide by NDST activity, diluted to 1 μg/ml (1:1000 dilution) with 0.2% gelatin in TBS-T was added. After an hour of incubation at room temperature, wells were washed with TBS-T, and 100 μL of an HRP-labeled goat anti-mouse immunoglobulin G+M antibodies [82], which were diluted with 0.2% gelatin in TBS-T (1:20,000 dilution), were added to each well. After another hour of incubation at room temperature, wells were washed again with TBS-T, and 100 μl/well of 3,5,3’,5’ tetramethylbenzidine (TMB, Thermo Scientific) substrate solution was added to develop the color. The reaction was stopped by adding 100 μl of 2M H_2_SO_4_ to each well. Absorbance was measured at 450 nm (reference wavelength 630 nm). Protein concentration was determined using the BCA assay. Assays were performed in triplicates.

### 35S-incorporation in MPS patient fibroblasts

GAG synthesis rate was determined by monitoring ^35^S incorporation in cultured fibroblasts from two MPS IIIA and one MPS IIIC patient. Cells were plated in a 12-well plate containing DMEM (Wisent, PQ, Canada) + 10% FBS and penicillin/streptomycin (Gibco) at 37°C in the presence of 5% CO_2_. The next day, the medium was supplemented with 1.2 μM (1xIC_50_) SAHA or DMSO only. After 5 days treatment, the medium was replaced with DMEM/F12 (Invitrogen) medium supplemented with 10% dialyzed FBS containing 10 μCi/ml H_2_[^35^S]O_4_ (PerkinElmer Life Science), and 1.2 μM SAHA or DMSO. To assess three time points of ^35^S-incorporation, 24 hr, 48 hr and 72 hr later, a set of cells (in triplicate) was washed six times with PBS and lysed in 0.1 N NaOH. ^35^S incorporation was measured in the cell lysates using a scintillation counter (Beckmann). Protein determination was measured in aliquots of the same cell lysates by BCA assay and used to normalize ^35^S counts. All experiments were performed in triplicates.

### Statistical analysis

Non-linear regression analysis and Student’s *t*-test were performed using GraphPad Prism. P values of < 0.05 were considered statistically significant.

## Results

### Analysis of *NDST1* promoter sequence

**Fig 1:**
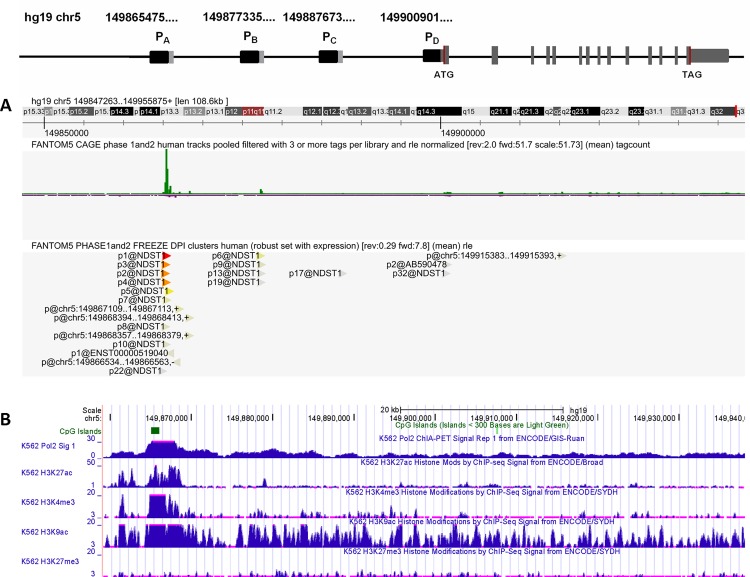
Schematic representation of *NDST1* gene. The human *NDST1* gene is located on chromosome 5 and encompasses 15 exons. The first exon and a part of the second are untranslated regions. *NDST1* contains four alternative transcription starting sites indicated as P_A_, P_B_, P_C_, and P_D_. (A) The parametric clustering of CAGE sequences (green peaks) and DPI clusters (red and yellow arrows) indicates high tag expression level for P_A_ region. (B) Transcription associated features such as CpG island, Pol II binding site and histone methylation/acetylation profile identifies P_A_ region as the most usable promoter for NDST1 gene transcription. Data is filtered for K562 cell line. Screenshots for NDST1 promoter analysis were obtained from ZENBU genome browser (FANTOM5 Project) (http://fantom.gsc.riken.jp/zenbu/) and from UCSC Genome Database browser (http://genome.ucsc.edu/) using Human Feb. 2009 (GRCh37/hg19) Assembly.

**Fig 2:**
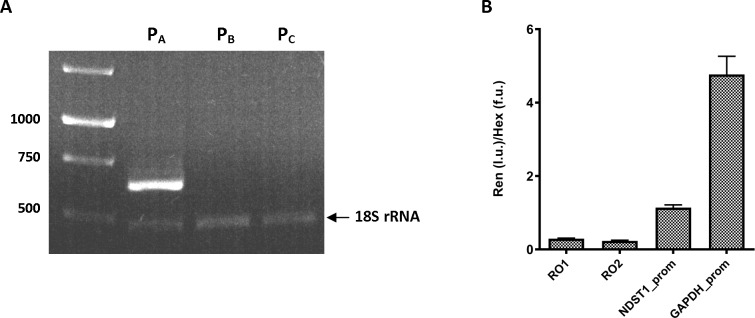
Confirmation of alternative *NDST1* transcripts by RT-PCR and assessment of *NDST1* promoter (P_A_ region)—luciferase construct activity. (A) Reverse transcription PCR for alternative *NDST1* transcripts. Complementary DNA reversely transcribed from total extracted RNA of normal human fibroblast was used in the PCR to identify expression levels of each of the possible *NDST1* transcripts. Primers flanked the sequence of 5’ UTR (exon 1) and coding region (exon 2) were expected to produce 637, 700, 688 bp PCR fragments for NDST1 mRNA transcribed from P_A_, P_B_ and P_C_ respectively. PCRs with these primers amplified only NDST1 mRNA transcribed from P_A_ region. 18S rRNA was used as an internal control. (B) Activity of *NDST1* promoter—renilla luciferase reporter construct. Renilla luciferase expression vector containing either: i) two random DNA sequences, RO1 and RO2 (negative controls); ii) *NDST1* promoter (P_A_ region); or iii) *GAPD*H promoter (positive control) were transiently transfected into HeLa cells. DNA fragments activity is shown as a normalized value of luminescence (produced by renilla luciferase) to fluorescence (produced by Hex). Experiments were conducted in triplicates. Ren—renilla luciferase activity, l.u.—luminescence units, f.u.—fluorescence units.

The human *NDST1* gene is located on chromosome 5 (q33.1). It is encoded by 15 exons having the first exon and a part of the second exon as an untranslated region. Based on the *NDST1* mRNA sequences submitted to the genomic databases, it is evident that *NDST1* is transcribed from several alternating transcription starting site. As indicated in Fig 1, transcription of *NDST1* can be initiated from P_A_, P_B_, P_C_, and P_D_ at ch5:149865475, 149877335, 149887673, and 149900901 respectively. To determine which of these promoters can be applied for our HTS, i.e. the promoter or promoters that are predominantly used for *NDST1* transcription, we evaluated the promoter supportive evidences using the cap analysis gene expression (CAGE) data generated by FANTOM5 project [14]. We have also analyzed alternative NDST1 promoter sequences submitted to the UCSC Genome Browser database for the promoter associated features such as RNA polymerase II (Pol II) binding site, CpG island, and histone acetylation or methylation [15]. The CAGE expression signal-based track from the sequencing of 1829 human samples shows two distinct CAGE expression peaks at ch5:149865475 (P_A_ region) and at ch5:149877335 (P_B_ region). Although, the expression level of *NDST*1 mRNA from P_A_ region is much higher than from P_B_ region, as indicated by pooled CAGE tracks and decomposition-based peak identifier (DPI) clusters (Fig 1A). its high transcriptional activity is also supported by the presence of CpG island, Pol II binding site, enrichment of the H3K27ac, H3K4me3, and H3K9ac signals and depletion of H3K27me3 signal (Fig 1B). In addition to *in silico* analysis of *NDST1* promoter regions we determined the expression level of alternatively spliced *NDST1* mRNAs in normal human fibroblast cells by performing reverse transcription-polymerase chain reaction (RT-PCR) analysis using unique forward primers for each alternative exon 1 and a common reverse primer for exon 2. Positive outcome of RT-PCR was expected to result in products of 637 bp for mRNA from P_A_, 700 bp for mRNA from P_B_, and 688 bp for mRNA from P_C_ regions respectively. In addition to *NDST1* primers, each reaction contained 18S rRNA primers to amplify a 484 bp region to serve as an internal positive control. It should be noted that we could not test for the expression level of a *NDST1* mRNA transcript originating from the P_D_ region since transcription of this mRNA would be initiated from the second exon and all four reported alternative *NDST1* mRNAs have same second exon. The RT-PCR of the alternative *NDST1* transcripts identified the efficient transcription of the *NDST1* mRNA from P_A_ region, whereas undetectable level of PCR product was obtained for *NDST1* mRNAs from P_B_ and P_C_ regions (Fig 2A). The identity of the amplified PCR product was confirmed by sequencing and resulted in 100% identity with the published sequence of the first exon for 209107 cDNA clone (http://genome.ucsc.edu/).

### *NDST1* promoter—luciferase reporter gene construct activity

We quantified and compared the activity produced by the *NDST1* promoter to that produced by two random DNA regions (negative controls) or the *GAPDH* promoter (a positive control) in transient transfections using HeLa cells. The ability of these DNA regions to drive the expression of renilla luciferase was assessed by a luminescence assay. Although the same expression vector (pLightSwitch_Prom) was used for all four DNA fragments, to avoid any inconsistency of transient transfections and to control for cell number variability we performed an additional assay (β-hexosaminidase, Hex assay) in parallel to the luminescence assay. From the normalized activity of the luminescence produced by firefly luciferase to the fluorescence produced by Hex, we determined that *NDST1* promoter activity was threefold higher than negative controls RO1 and RO2, and approximately fivefold lower than the positive control *GAPDH* (Fig 2B). While the *NDST1* promoter was not as active as the *GAPDH* promoter, its activity was sufficient for HTS.

To exclude any potential transient transfection variations, we chose to perform HTS using a clonal permanent HeLa cell line stably expressing *NDST1* promoter. The initial *NDST1* promoter—renilla luciferase construct used in transient transfections could not be used to generate a permanent cell line as it did not contain an appropriate antibiotic selection marker; therefore we sub-cloned *NDST1* promoter from pLightSwitch_Prom into pGL4.22 [luc2CP/Puro] expression vector by restriction digestion. The integrity of a newly produced *NDST1* promoter—firefly luciferase construct was validated by sequencing. After transfection of HeLa with *NDST*1 in pGL4.22 [luc2CP/Puro], several stable cell colonies were validated for the amount of the luminescent light generated and a cell colony with the highest luminescence activity (~500,000 relative luminescence units) was selected for HTS (data not shown).

### High-content screening (Primary screen)

We validated our firefly assay for reliability and robustness by determining the distribution of the luminescence produced by HeLa cells stably expressing firefly luciferase under control of *NDST1* promoter. From the treatment of one 96-well plate of cells with DMSO only, the mean of luminescence was calculated as 235,428 luminescence units and the standard deviation from the mean of DMSO was 20,494.

**Fig 3:**
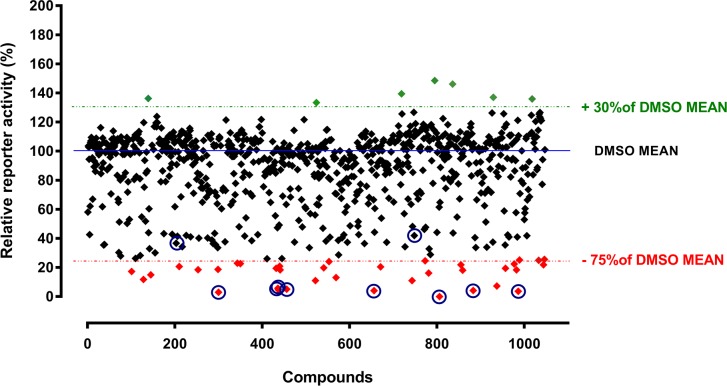
High-content screening of 1,200 Prestwick Chemical Library compounds. HeLa cells stably expressing firefly luciferase under control of *NDST1* promoter were treated with compounds from the Prestwick Chemical Library. The reporter activity of the tested compounds is expressed relative to the mean activity of DMSO treated cells. Drugs showing cytotoxic effects, as tested in parallel by the Hexosaminidase assay, were removed from the graph. Thirty eight compounds resulting in the luminescence decrease by >75% relative to the mean of DMSO control were considered as potentially strong *NDST1* inhibitors (shown in red). Seven compounds (shown in green) increased luminescence by ≥ 30% over the mean DMS control. These compounds could be considered as potential inducers of *NDST1* expression. Eight potentially strong inhibitors and 2 average inhibitors (indicated by blue circles) were selected for the dose-response assessment (Fig 4A). Experiments were performed in triplicates. Abbreviation: stdev–standard deviation.

After validation of the firefly luciferase assay, we screened 1,200 compounds from the Prestwick Chemical Library. For all plates we used DMSO as a negative control. Also, we performed the lysosomal Hex assay in parallel with the firefly luciferase assay to control for cytotoxic effect of the compounds. Cells with the survival rate of less than 75% were excluded from further analysis (data not shown). Results of the primary screen, with exclusion of the cytotoxic compounds, are represented in Fig 3. We defined compounds as strong inhibitors, when decreasing *NDST1* promoter activity by 75–100%, as average inhibitors, when decreasing *NDST1* promoter activity by 50–75%, or as inducers, when increasing *NDST1* promoter activity by at least 30% or more relative to the mean of DMSO control. We identified 38 compounds as potentially strong inhibitors (S1 Table) and 7 compounds as potentially strong inducers of *NDST1* promoter activity. Based on the chemical structure similarity we divided inhibitory compounds into three groups: i) 17 compounds containing steroid back-bone structure; ii) 10 compounds containing pyridine or imidazole ring structures, and iii) 11 compounds containing unique structures.

### Secondary screening–‘Hit’ validation

From a list of potential repressors of *NDST1* promoter activity obtained from the primary screen, we selected 8 compounds with the highest inhibitory score above 75% and 2 other compounds with the inhibitory score between 75% and 50% for further analysis (Table 2). We selected the two latter compounds because one of these, pyrimethamine, was previously identified in another one of our HTS campaigns [16]. Pyrimethamine has been shown to act as an EET- agent for mutant forms of β-hexosaminidase resulting in juvenile or adult GM2 gangliosidosis and was successfully tested it in Phase I/II clinical trial [16]. Also, as a part of our previous work, we obtained a set of novel derivatives of the molecule from Marco Ciufolini (University of British Columbia) that we could also test if pyrimethamine proved to be a suitable candidate in secondary screens. The second compound, griseofulvin (58% reduction), was picked as a random control to see whether compounds inhibiting *NDST1* reporter activity by ~50% were generally worthy of additional testing.

**Table 2:** The eight highest score and two mid-range score NDST1 suppressors selected for secondary screening.

Plate Well	Compound	% Inhibition	PubChem CID	CAS Number	Mechanism
03E11	Fenbendazole	79.34	3334	43210-67-9	Microtubule formation inhibitor
04F10	Mifepristone	81.24	55245	84371-65-3	Progesterone receptor antagonist
06F07	Meclocycline sulfosalicylate	94.85	5282520	73816-42-9	Ribosomal protein synthesis inhibitor
07E03	Thiostrepton	89.02	16220061	1393-48-2	n/a
07H04	Pentamidine isethionate	75.87	6604151	140-64-7	n/a
08A10	Entacapone	86.87	5281081	130929-57-6	catechol-O-methyl transferase inhibitor
14C11	Vorinostat (SAHA)	92.38	5311	149647-78-9	histone deacetylase (HDAC) inhibitor
15D10	Anthralin	78.3	10187	1143-38-0	antipsoriatic
01D08	Pyrimethamine	64.44	4993	58-14-0	Folic acid antagonist
03G07	Griseofulvin	57.9	441140	126-07-8	Enzymatic inductor

While luciferase based assays are becoming increasingly used in gene expression studies, false positives may occur if firefly luciferase directly interacts with any of the compounds from the chemical libraries. The assessment of a large set of compounds from the NIH Molecular Libraries Small Molecule Repository has shown that the activity of firefly luciferase could be altered due to its direct binding to 12% of the compounds [17]. To determine if any of our best candidates represented such false positives, we performed an *in vitro* binding luciferase assay using cell lysates containing firefly luciferase in the presence of each potential inhibitor identified from the screen. We found that half of tested compounds had almost no effect (7–15% decrease) and the other half had moderate effect (26–42% decrease) on the firefly luciferase activity. It should also be noted that these values represent the maximum treatment effect, as the amount of the compound added to cell lysate likely exceeds the amount of the compound internalized by the cells used in HTS.

**Fig 4:**
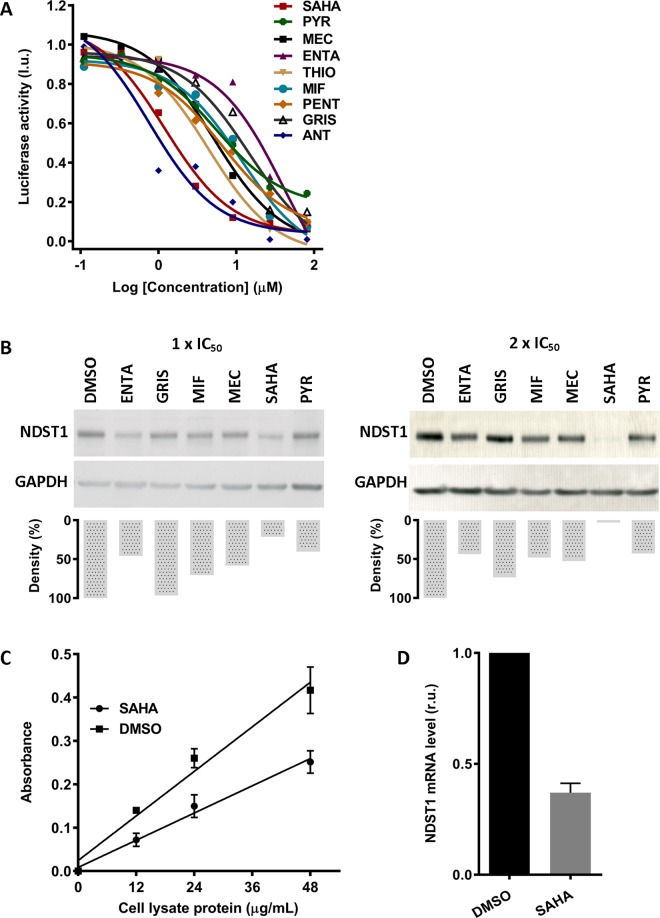
Secondary screening and ‘hit’ validation. (A) Dose-dependent inhibition of the *NDST1* promoter activity. HeLa cells stably expressing *NDST1* promoter were treated with serially diluted potential *NDST1* repressors. Dose-response curves were produced from 9 out of 10 drug candidates. Experiments were performed in singles. (B) Western blot densitometry analysis of NDST1 protein expression after treatment with selected potential *NDST1* repressors. Normal human fibroblast cells were treated with potential NDST1 inhibitors at their calculated 1x IC_50_ or 2x IC_50_. The effect of the compound on the NDST1 expression was evaluated using densitometry analysis of NDST1 band normalized to its corresponding GAPDH band. Distinct decrease in *NDST1* expression was observed for 2x IC_50_ SAHA treatment. (C) ELISA assay of total NDST activity in normal human fibroblast cells. Lysates of cells treated with either SAHA or DMSO were assessed for total NDST activity. Treatment with 1.2 μM of SAHA resulted in a ~ 40% decrease in NDST enzyme activity. Experiments were conducted in triplicates. (D) The effect of SAHA on *NDST1* mRNA expression in normal human fibroblast cells. Normal human fibroblast cells were treated with 2x IC_50_ (2.4 μM) of SAHA for 5 days. The level of *NDST1* mRNA was reduced by 60% after the treatment. Expression of *NDST1* mRNA level was normalized to 18S rRNA level. Experiments were performed in duplicates. MEC–meclocycline, THIO–thiostrepton, ENTA–enthacapone, MIF–mifepristone, SAHA–SAHA, GRIS–griseofulvin, ANT–antralin, PENT–penthamidine, PYR–pyrimethamine. r.u.–relative units, l.u.–luminescence units.

In order to examine a dose-response of selected NDST1 inhibitors from the primary screen we treated HeLa cells stably expressing firefly luciferase under control of NDST1 promoter with serially diluted compounds. Toxicity of the compounds was monitored by a total protein concentration in each sample. Using data analysis outlined in Materials and Methods, we calculated IC_50_ for each tested compound. Dose-response curves were produced for 9 out of 10 hits, i.e. fenbendazole did not produce a curve from which an IC_50_ could be calculated (Fig 4A). Total protein concentration in each sample remained consistent over the treatment.

The effect of the tested compounds from the dose-response experiments were further validated in a normal human fibroblast cell line. Cells were treated with each compound at their calculated 1x IC_50_ and 2x IC_50_ for five days. During the treatment, four compounds fenbendazole, anthralin, apomorphine hydrochloride hemihydrates and penthamidine isetionate were found to be cytotoxic to cells and were excluded from further analysis. Treated cells with the remaining six compounds mifepristone, meclocycline sulfosalicylate, entacapone, SAHA, pyrimethamine, and griseofulvin were harvested and their cell lysates were analyzed by Western blot using specific NDST1 antibodies. The effect of the compounds on NDST1 expression was validated by densitometry of normalized NDST1 protein levels (Fig 4B).

After treatment with selected compounds, we found more than 50% reduction in NDST1 expression in normal human fibroblast cells treated with 1x IC_50_ or 2x IC_50_ of enthacapone, SAHA, and pyrimethamine. The strongest response was obtained for SAHA treatment; at 2x IC_50_ (2.4 μM), SAHA reduced expression of NDST1 to nearly undetectable level. By contrast, there was almost no response observed in cells treated with griseofulvin, our randomly selected low-inhibition control. Also, low response was observed for pyrimethamine, our second low-inhibition control.

### Further evaluation of the top ‘hit’, SAHA

We selected SAHA as our best SRT candidate, as it significantly reduced endogenous expression of NDST1 at low concentrations. To evaluate if the observed effect of SAHA on NDST1 expression in normal human fibroblast cells also correlated with a decrease in total endogenous NDST enzyme activity, we utilized an ELISA-based assay to determine NDST activity before and after treatment. After treatment of cells with 1 x IC_50_ (1.2 μM) of SAHA for five days, cell lysates were incubated with immobilized, biotin labeled K5 (N-acetyl heparosan) or with DMSO alone. The total N-deacetylase activity of NDST (1–4) was determined by using an antibody specific for deacetylated form of heparosan (3’-phosphoadenosine 5’-phosphosulphate was not present in the assay, thus N-sulfate could not occur). After this treatment we found that SAHA was able to decrease the total activity of NDSTs by approximately 40% (Fig 4C).

We also evaluated the effect of SAHA on the level of *NDST1* mRNA in normal human fibroblast cells. After five days treatment with SAHA at 2x IC_50_ (2.4 μM), extracted RNA from cells was analyzed for the expression of *NDST1* gene by qPCR analysis. The level of amplified fragments in each reaction was normalized to the level of 18s rRNA. Quantification of relative gene expression was calculated using the 2^–DDC^_T_ (Livak) method. All qPCR reactions had amplification efficiencies between 95–99% and were performed in duplicates. After treatment, we detected a significant reduction in *NDST*1 mRNA level to 0.37 relative units (relative expression normalized to 1) (Fig 4D).

**Fig 5:**
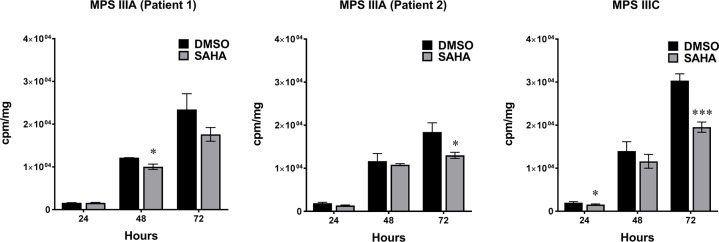
^35^S Incorporation into newly synthesized GAGs. After five days of SAHA treatment, incorporation of ^35^S into GAGs was measured at 24, 48 and 72 hours of pulse. All patient cells showed reduced level of ^35^S labeled GAGs synthesis. Experiments were conducted in triplicates. cpm–counts per million, mg–milligrams of protein. * p < 0.05, *** p < 0.001.

To determine the effect of SAHA on the synthesis of sulfated GAGs in MPS patient cells we performed a radioactive ^35^S incorporation analysis. Since GAGs are the predominate macromolecules produced by fibroblasts that incorporate inorganic ^35^S [18], we grew cells in media containing H_2_[^35^S]SO_4_ to measure incorporation of ^35^S into newly synthesized GAGs. After five days SAHA treatment and 72 hours of the pulse, incorporation of ^35^S in MPS IIIA (patient 1), MPS IIIA (patient 2), and MPS IIIC cell lines was reduced by 25%, 30%, and 36%, respectively (Fig 5).

### Histone deacetylase inhibitors

**Fig 6:**
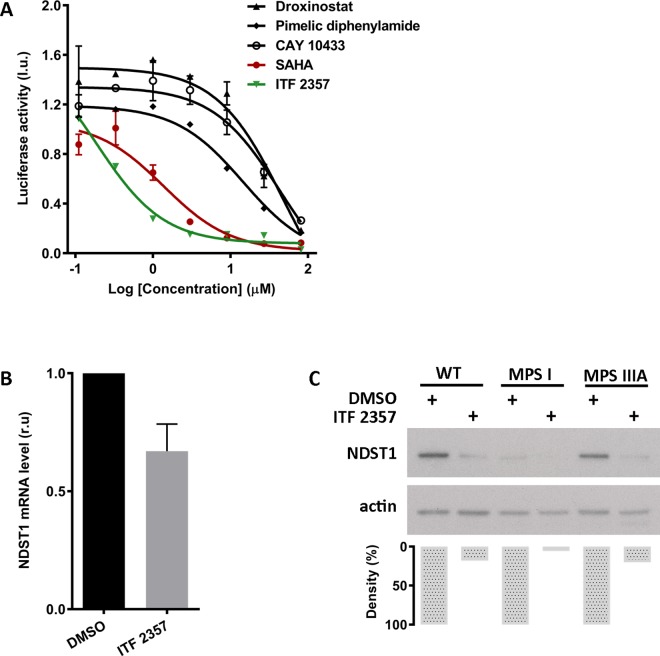
Histone deacetylase (HDAC) inhibitor assessment. (A) Dose-response of HDAC inhibitors on *NDST1* promoter. HeLa cells stably expressing the *NDST1* promoter—luciferase reporter gene were treated with serially diluted selected HDAC inhibitors. ITF 2357, an HDAC inhibitor which is structurally and functionally similar to SAHA, produced a dose-response curve with IC_50_ of 0.2 μM. Experiments were performed in duplicates. (B) *NDST1* mRNA level after treatment with ITF 2357. Normal human fibroblast cells were treated with 2x IC_50_ (0.4 μM) of ITF 2357 for 5 days. Level of NDST1 mRNA was reduced by 40% after the treatment. Expression of NDST1 mRNA level was normalized to 18S rRNA level. Experiments were conducted in duplicates. (C) Western blot densitometry analysis of NDST1 protein expression after treatment with ITF 2357. Normal human fibroblast, MPS I patient, and MPS IIIA patient cells were treated with ITF 2357 at its calculated 2x IC_50_. The effect of the compound on the NDST1 expression was evaluated using densitometry analysis of NDST1 band normalized to its corresponding actin band. Significant decrease in NDST1 expression was observed in all treated cells. l.u.–luminescence units. r.u.–relative units.

SAHA is a potent histone deacetylase (HDAC) inhibitor that modulates expression of genes involved in cell signaling and cell growth. Since SAHA inhibits both class I and class II HDACs, we tested other known HDAC inhibitors in an attempt to elucidate the mechanism by which SAHA affects *NDST1* transcription. If SAHA acts on *NDST1* expression through its histone deacetylase activity, then other HDAC inhibitors should have analogous effect on *NDST1* expression. We selected four inhibitors for a dose-response assessment: droxinostat, explicit for HDACs 3, 6, and 8; pimelic diphenylamide, specific for class I HDACs; CAY 10433 and ITF 2357, specific for class I and II HDACs. The latter HDAC inhibitor, ITF 2357, is structurally highly comparable to SAHA. Similar to the dose-response assessment for NDST1 inhibitors from the primary screen, HeLa cells stably expressing firefly luciferase under control of NDST1 promoter were treated with serially diluted HDAC inhibitors. Out of four tested compounds only ITF 2357 showed a better response with IC_50_ of 0.2 μM compared to IC_50_ of 1.2 μM for SAHA (Fig 6A). When tested by qPCR, the level of *NDST1* mRNA in normal human fibroblast cells was reduced by 40% after five days treatment with 2x IC_50_ of ITF 2357 (Fig 6B). In addition, Western blot analysis showed reduction in NDST1 protein level in normal human fibroblast and MPS IIIA patient cells by 80% and in MPS I patient cells by 95% after 1 day with 2x IC_50_ of ITF 2357 treatment (Fig 6C).

## Discussion

Previously published data indicate that NDST activity is a key-controlling factor in the degree of modification that occurs to the HS precursor producing S-domains. In spite of having similar structures, the NDST isozymes (1–4) vary significantly in their tissue expression levels, and their N-deacetylation and N-sulfation activities [11]. Various studies suggest that NDST1 is the most important isozyme in terms of initiating the various HS modifications (see Introduction). Additionally, elevated levels of accumulated HS within lysosomes have been shown to increase the expression of HS sulfotransferases and epimerases [19], as well as the activity of NDST isozymes [20]. Over-activation of these enzymes will eventually result in a positive-feedback loop that ultimately produces higher levels of hyper sulfated HS with increase occurrences of iduronic acid residues (S-domains), thereby increasing the rate and size of MPS-fragment storage in some MPS patients tissues.

In this study we examined the hypothesis that by decreasing NDST1 activity through transcriptional inhibition, we could reduce the size and frequency of S-domains in the mature HS polysaccharide chains of MPS patient cells. This would reduce the levels of HS degrading enzymes; such as α-L-iduronidase, iduronate-2-sulphatase, heparan-N-sulphatase, acetyl CoA:N-acetyltransferase, N-acetylglucosamine-6-sulphatase, arylsulfatase G; needed to prevent or reduced storage in patients with either of 6 different types of MPS disorders. To test this hypothesis in the most efficient manner we used a luciferase reporter construct whose expression was driven by a minimal *NDST1* promoter fragment. In order to produce this construct we first had to identify the transcription initiation site in the *NDST1* DNA (Figs 1 and 2A) and confirm that the corresponding promoter fragment, available from SwitchGear Genomics, was indeed functional (Fig 2B). Hela cells were stably transfected with the luciferase reporter construct driven by this promoter fragment and confirmed to generate sufficient enzyme activity to allow a HTS campaign to be conducted (Fig 3). Various hits from the screen were tested in secondary assays and several were confirmed to reduce luciferase activity, likely by directly inhibiting transcription from the reporter construct. The ability of these compounds to inhibit the transcription of *NDST1* from its endogenous promoter was then examined by Western blotting (Fig 4B). The most potent ‘hit’ identified, SAHA, was further confirmed as a transcriptional inhibitor of *NDST1* by direct enzyme activity measurements of total NDST (Fig 4C), and qPCR of NDST1 mRNA in treated versus untreated normal human fibroblasts (Fig 4D). Finally, we demonstrated that SAHA treatment could significantly decrease the incorporation of ^35^S GAGs in patient fibroblasts (Fig 5)

The above results from cells treated with SAHA could suggest that it affects NDST1 expression through its deacetylase activity. Thus we tested other HDAC inhibitors (Fig 6). Of those tested ITF 2357 was also found to be a strong inhibitor of the reporter construct (Fig 6A) and of endogenous *NDST1*expression, i.e. that ITF 2357 could also reduce *NDST1* mRNA and protein levels in fibroblasts (Fig 6B and 6C). However, both SAHA and ITF 2357 have similar chemical structures and thus inhibition could also be a structure dependent effect that is not related to their known HDAC inhibitor activity. This hypothesis was consistent with our observation that CAY 10433 treatment produced only a low level of inhibition in the HeLa cells reporter assay (Fig 6A), even though it also inhibits all class I and class II HDACs.

A proof of principal for the effectiveness of decreasing HS biosynthesis as an approach to SRT in the MPSs has been published. When the activity of Ext1 and 2 (the elongation isoenzymes in the first steps of HS biosynthesis) were reduced by as little as 30–50%, the amount of disease-specific biomarker and pathology in multiple tissues, including the brain in MPS IIIA mice, were ameliorated [21]. In this study a decrease in Ext activity and thus SRT, was achieved genetically by cross breading MPS IIIA mice with other asymptomatic mice heterozygous for deletions of either their *Ext1* or *Ext2* genes. The results suggest that complete normalization of lysosomal storage may not be necessary to correct pathology in the brain and that small reductions in HS biosynthesis might be sufficient to elicit these effects [21]. This report also demonstrated that this SRT approach could improve the efficacy of ERT in cell culture and in mice. Finally, the report demonstrated that the reduction in HS biosynthesis by as much as 50% produces no detrimental secondary effects in any of the groups of mice used in the study.

Our data confirm that the targeting of *NDST1* expression has a strong potential to provide a basis for SRT in some MPS patients; adding a needed therapeutic strategy that could serve as a standalone or adjunct, e.g. along with ERT, therapy. By extension it should also improve the efficacy of any form of gene therapy that may emerge in the future for these disorders.

In this study we used a reporter construct driven by a small *NDST1* promoter fragment, which likely contained only a small subset of the regulator elements of the full promoter. However, our goal here was to provide a proof of principal for our target, which we accomplished. The next step in identifying more diverse and potent transcriptional inhibitors, that could be used singly or in combinations, will be to produce a cell line in which we have placed the reporter luciferase cDNA directly in front of the endogenous *NDST1* gene’s transcription initiation site, which we identified (Figs 1 and 2), using the new CRISPR/Cas9 technology, and to repeat our HTS using a more extensive drug library.

## Supporting Information

S1 Table Potential strong inhibitors of the NDST1 promoter activity. (DOCX)
