# Supplementary material for: NDST1 Preferred Promoter Confirmation and Identification of Corresponding Transcriptional Inhibitors as Substrate Reduction Agents for Multiple Mucopolysaccharidosis Disorders
Source: PLoS One. 2016 Sep 22;11(9):e0162145. doi: 10.1371/journal.pone.0162145 (PMC5033324; doi:10.1371/journal.pone.0162145)
Supplement: S1 Table — (DOCX) [file pone.0162145.s001.docx]

S1 Table. Potential strong inhibitors of the NDST1 promoter activity.

| **No** | **Plate Well** | **Compound** | **Structure** | **% Inhibition** |
| --- | --- | --- | --- | --- |
| 1 | 02C02 | R(-) Apomorphine hydrochloride hemihydrate |  | 82.66 |
| 2 | 02E09 | Spironolactone |  | 88.19 |
| 3 | 02G06 | Chlortetracycline hydrochloride |  | 84.98 |
| 4 | 03E11 | Fenbendazole |  | 79.34 |
| 5 | 04B04 | Norethindrone |  | 81.5 |
| 6 | 04F10 | Mifepristone |  | 81.24 |
| 7 | 04F11 | Diperodon hydrochloride |  | 97.00 |
| 8 | 05C04 | Desipramine hydrochloride |  | 76.96 |
| 9 | 05C11 | Clozapine |  | 77.38 |
| 10 | 06D03 | Corticosterone |  | 80.55 |
| 11 | 06D07 | Digitoxigenin |  | 94.22 |
| 12 | 06D08 | Digoxin |  | 95.27 |
| 13 | 06D11 | Epiandrosterone |  | 79.08 |
| 14 | 06E02 | Estradiol-17 beta |  | 81.64 |
| 15 | 06F07 | Meclocyclinesulfosalicylate |  | 94.85 |
| 16 | 07E03 | Thiostrepton |  | 89.02 |
| 17 | 07G02 | Ciclopirox ethanolamine |  | 80.22 |
| 18 | 07H04 | Pentamidineisethionate |  | 75.87 |
| 19 | 08A10 | Entacapone |  | 86.87 |
| 20 | 09B07 | Lanatoside C |  | 95.92 |
| 21 | 09D02 | Dydrogesterone |  | 79.54 |
| 22 | 10C04 | Medrysone |  | 89.00 |
| 23 | 10F04 | Norgestrel-(-)-D |  | 75.33 |
| 24 | 10G02 | Clobetasol propionate |  | 83.79 |
| 25 | 11F06 | Beclomethasonedipropionate |  | 78.05 |
| 26 | 11F10 | Fluvastatin sodium salt |  | 81.83 |
| 27 | 12A04 | Digoxigenin |  | 95.78 |
| 28 | 12F08 | Dehydroisoandosterone 3-acetate |  | 92.74 |
| 29 | 12H08 | Deoxycorticosterone |  | 80.47 |
| 30 | 13B08 | Zardaverine |  | 77.54 |
| 31 | 13C03 | Nandrolone |  | 81.65 |
| 32 | 13C07 | Proscillaridin A |  | 96.32 |
| 33 | 13H04 | Nomegestrol acetate |  | 75.00 |
| 34 | 14A05 | Prednicarbate |  | 78.14 |
| 35 | 14C11 | Vorinostat (SAHA) |  | 92.38 |
| 36 | 15F03 | Atorvastatin |  | 87.31 |
| 37 | 15E05 | Ethinylestradiol |  | 90.7 |
| 38 | 15D10 | Anthralin |  | 78.3 |
